# Neurobiology, Pathophysiology, and Treatment of Melatonin Deficiency and Dysfunction

**DOI:** 10.1100/2012/640389

**Published:** 2012-05-02

**Authors:** Rüdiger Hardeland

**Affiliations:** Johann Friedrich Blumenbach Institute of Zoology and Anthropology, Georg August University, 37073 Göttingen, Germany

## Abstract

Melatonin is a highly pleiotropic signaling molecule, which is released as a hormone of the pineal gland predominantly during night. Melatonin secretion decreases during aging. Reduced melatonin levels are also observed in various diseases, such as types of dementia, some mood disorders, severe pain, cancer, and diabetes type 2. Melatonin dysfunction is frequently related to deviations in amplitudes, phasing, and coupling of circadian rhythms. Gene polymorphisms of melatonin receptors and circadian oscillator proteins bear risks for several of the diseases mentioned. A common symptom of insufficient melatonin signaling is sleep disturbances. It is necessary to distinguish between symptoms that are curable by short melatonergic actions and others that require extended actions during night. Melatonin immediate release is already effective, at moderate doses, for reducing difficulties of falling asleep or improving symptoms associated with poorly coupled circadian rhythms, including seasonal affective and bipolar disorders. For purposes of a replacement therapy based on longer-lasting melatonergic actions, melatonin prolonged release and synthetic agonists have been developed. Therapies with melatonin or synthetic melatonergic drugs have to consider that these agents do not only act on the SCN, but also on numerous organs and cells in which melatonin receptors are also expressed.

## 1. Introduction

Melatonin (*N*-acetyl-5-methoxytryptamine) has been discovered as a hormone of the pineal gland, but it is meanwhile known to be also synthesized in various other organs, tissues, and cells [1–3]. Pineal melatonin is secreted, in young and middle-aged individuals, in a circadian fashion, with a high rhythm amplitude and a prominent nocturnal maximum. Melatonin from extrapineal sites often oscillates with considerably lower amplitudes. Some of the extrapineal sources are, according to current knowledge, of particular importance, either in quantitative terms, such as the gastrointestinal tract (GIT), which contains several hundred times more melatonin than the pineal gland [1–6], or, with regard to functional aspects, some areas of the central nervous system (CNS) [3, 7] and several leukocytes [1, 3, 8, 9]. The physiological significance of most other sites of melatonin biosynthesis is poorly understood. Melatonin is not secreted in substantial amounts from most of the extrapineal sites, or only under specific conditions. A well-known example is its postprandial release from the GIT, during which relatively high quantities can enter the circulation, where they remain only for a short period of time and are chronobiologically rather irrelevant [5, 6, 10, 11]. Thus, melatonin is not only a pineal hormone but also has additional functions as a local tissue factor and leukocyte-derived cell hormone with paracrine and autocrine actions [12]. Moreover, melatonin shows properties of a powerful antioxidant, at sufficiently high concentrations as a direct radical scavenger [13–15], but, at lower, near-physiological levels, as a regulator of redox-relevant enzymes [1, 6, 15, 16], suppressor of prooxidant excitatory and inflammatory processes [3, 6, 16–19] and as a mitochondrial modulator [18–25]. With regard to the pathophysiological importance of mitochondrial dysfunction [18, 24, 26–29] and to reports on intramitochondrial accumulation of melatonin [25, 30], it may be suggestive to assume a particular relevance of especially this latter property.

 The functional complexity of melatonin's actions extends to the multiplicity of target cells [3] and to signal transduction mechanisms [31], which are much more diverse than originally believed. In humans, a classic site of action is the hypothalamic circadian pacemaker, the suprachiasmatic nucleus (SCN). This structure is a major site at which melatonin exerts its chronobiotic, that is, phase shifting, and its sleep-inducing effects. However, neither circadian phase shifts nor sleep initiation are exclusively restricted to the SCN. Findings on melatonin-induced changes in peripheral circadian oscillators have been recently summarized [32]. Sleep induction by melatonin also comprises thalamic effects that are detectable in the promotion of sleep spindles [33–35]. Nevertheless, the presence of particularly high densities of membrane-bound, G-protein-coupled melatonin receptors (GPCRs) in the SCN strongly supports the premier significance of this structure in receiving and transmitting the melatonin signal. In mammals, two melatonin-binding GPCRs exist, MT_1_ (= Mel_1a_ = MTNR1A) and MT_2_ (Mel_1b_ = MTNR1B), and both of them are present in the SCN of most species. In the human SCN, however, MT_2_ seems to be either absent or only expressed at very low levels [3]. Although MT_2_ is mainly responsible for phase shifting in other mammals, the human circadian master oscillator is reset by melatonin [36, 37], which may be explained by the observation that both receptors can, to some degree and in some organs, mutually substitute for each other [3, 31]. Outside the SCN, MT_1_ and/or MT_2_ receptors have been detected in numerous tissues, such as other areas in the CNS, pituitary, duodenum, colon, caecum and appendix, gallbladder epithelium, parotid gland, exocrine pancreas, *β* cells of endocrine pancreas, breast epithelium, myometrium, placenta, granulosa and luteal cells, kidney (fetal), cardiac ventricular wall, aorta, coronary and cerebral arteries and other parts of peripheral vasculature, brown and white adipose tissues, platelets, and various immune cells (summarized in [3]). Additionally, other binding sites, in particular, presumed nuclear receptors, such as the splice variants *a*, *b,* and *d* of ROR*α* (retinoic acid receptor-related orphan receptor-*α*) and ROR*β* (= RZR*β*), are widely distributed [3, 38]. ROR*α* is almost ubiquitously expressed, with particularly high levels in T- and B-lymphocytes, neutrophils, and monocytes, whereas ROR*β* is found in brain, pineal gland, retina, and spleen. For other binding sites related to calcium-dependent metabolism, mitochondria, and detoxification of aromates, see [3, 31]. Via its receptors, melatonin exerts effects on the secretion of various other hormones, either directly or indirectly by influencing the circadian multioscillator system [32]. Moreover, countless publications have demonstrated antioxidant actions in numerous tissues, which may, in the experimental settings, be partially receptor independent. As far as they concern effects on antioxidant enzymes and mitochondrial function, they should be largely explained by receptor-dependent regulation mechanisms that can be activated at physiological melatonin concentrations.

 The remarkable pleiotropy of this methoxyindole, being a regulator of numerous functions in many tissues and cells, indicates that melatonin deficiency or dysfunction of melatonin signaling leads to a plethora of consequences, which go far beyond sleep difficulties. Results on receptor knockouts in animal experiments and human gene polymorphisms of MT_1_, MT_2_, and melatonin biosynthetic enzymes indicate an association of melatonin deficiency and dysfunction with numerous diseases, as different as forms of cancer, metabolic syndrome, diabetes type 2, rheumatoid arthritis, and various mood disorders (summarized in [32]).

## 2. Age- and Disease-Related Melatonin Deficiency

Melatonin deficiency has been mainly investigated in the pineal gland, circulation, saliva, cerebrospinal fluid, and, by measuring the metabolite 6-sulfatoxymelatonin, urine. The nocturnal melatonin peak is regularly observed to decrease during aging, though with considerable interindividual variability [39–44]. In aged individuals, the nighttime values are frequently almost indistinguishable from those obtained during daytime. However, daytime values can decrease during aging as well. As long as a melatonin rhythm is detectable, the nocturnal peak is frequently phase advanced in the elderly relative to young individuals [43]. All these changes can be caused by an aging-related deterioration of the circadian pacemaker or of the neuronal transmission to the pineal, similar to that observed in neurodegenerative disorders [43–46], or may result from pineal calcification [41].

Decreased levels of melatonin, which exceed those observed during normal aging, have been repeatedly described in neurodegenerative disorders, especially in Alzheimer's disease and other types of senile dementia [47–52]. In many affected individuals, the melatonin rhythm is practically abolished. These declines seem to be, in many cases, the consequence of SCN degeneration. Tissue destruction in the SCN or in the pineal gland that leads to reduced melatonin secretion and to sleep disturbances has been also observed in other cases. This was reported, for example, for juvenile hamartomas, which cause precocious puberty [53], and for craniopharyngiomas [54–56].

However, decreases in melatonin levels have been also reported to occur in many diseases and disorders without apparent SCN degeneration or destruction. These cases, which may appear unexpected from a mechanistic point of view, include neurological and metabolic conditions, such as diabetes type 2 and general insulin resistance, migraine and other forms of severe pain, and also certain types of cancer [57–80] (Table 1). For further details, see [3, 32].

## 3. Light-Induced Suppression of Melatonin

Health problems may also arise in conjunction with nocturnal light, which is particularly relevant in shift work. Nocturnal illumination is known to acutely suppress melatonin formation and secretion by the pineal gland [81], an effect that should not be confused with the perturbation of the circadian system [40, 82]. In addition to numerous preclinical investigations, studies in humans have addressed the roles of light intensity, duration, and spectral properties [83–87]. Moderately reduced levels of melatonin or 6-sulfatoxymelatonin have been reported to occur in the arctic [88, 89] and antarctic summer [90, 91]. However, their rhythms are maintained, but sometimes phase shifted. Apart from an assumed relation to an increased frequency of migraine [92], these changes do not seem to be of higher pathological relevance.

Particularly blue light is highly effective in suppressing melatonin, which is explained by the spectral sensitivity of melanopsin-containing retinal ganglion cells. These transmit, in parallel with a smaller contribution by green-absorbing cones, the photic information to the SCN [93–95] and from there to the pineal gland. In shift workers, the transient melatonin deficiency induced by nocturnal light is not compensated during later sleep phases for reasons of temporal position of the circadian clock. The consequences of reductions in the levels of the pineal hormone will be discussed in following sections.

## 4. Dysfunction of Melatonergic Signal Transduction

In humans, the evidence for disease-associated dysfunction of melatonin signaling is mainly based on changes in receptor densities or receptor polymorphisms. Decreased receptor expression can, of course, be the consequence of degenerative, in particular, neurodegenerative processes. In patients with Parkinson's disease (PD), MT_1_ and MT_2_ expression declines especially in the substantia nigra and the amygdala [96]. Decreases in MT_1_ and MT_2_ densities were observed in cerebral cortex and pineal gland of patients with Alzheimer's disease (AD) [97]. They also showed reduced expression of MT_2_ in hippocampus [98] and retina [99], and of MT_1_ in the SCN [46, 100]. However, MT_1_ density was reported to increase in pyramidal neurons of the hippocampal layers C1–C4 [101]. Whether or not this reflects a compensatory action, and to what extent this is associated with differences in signal transduction mechanisms (cf. [31]), remains to be clarified. Increases in MT_1_ expression were also observed in the cerebrovascular system, especially in intrahippocampal arteries [102]. Since MT_1_ and MT_2_ exert opposite actions in the vascular system, that is, activation of MT_1_ causing vasoconstriction, MT_2_ vasodilation [103–105], an imbalance between MT_1_ and MT_2_ signaling may indicate a dysregulation of cerebral blood flow [44, 102]. Indeed, lowered cerebral perfusion and hemodynamic microcirculatory insufficiency have been discussed as part of the AD pathophysiology [106] and may be induced by rises in MT_1_ expression.

Progressive reductions in receptor densities can be interpreted as a consequence of degeneration. Nevertheless, they may contribute to the severity of the disease. In AD, these alterations are accompanied by an SCN dysfunction, which also leads to a gradual disconnection of the pineal gland from its major input [45]. Moreover, the ongoing loss of melatonin receptors in late AD stages reduces the chance of alleviating some AD symptoms, such as sundowning and disturbed sleep, by melatonergic treatment, which is moderately possible in earlier stages [107–109].

 Impaired melatonin signaling may be also deduced from polymorphisms of melatonin receptors. A large body of evidence for an association of MT_2_ variants with a risk for diabetes type 2 has meanwhile accumulated (recently reviewed in [32]). Although the disease is multifactorial and the polymorphism only indicates a statistical probability, these findings shed light on the importance of melatonin in the maintenance of optimal functioning and health. Moreover, this view is supported by changes in insulin secretion, as observed in MT_2_ variants [110], and by corresponding results from animal models, including receptor knockouts [3, 32] and effects of melatonin on the PKC*ζ*/Akt/GSK3*β* signaling pathway [111].

 MT_2_ polymorphisms have been also related to rheumatoid arthritis [112] and, in combination with other risk factors, to adolescent idiopathic scoliosis [113], findings that may require further support by additional studies (cf. [32]). MT_1_ polymorphisms have been associated with polycystic ovary syndrome, including secondary effects on plasma glucose and insulin resistance [114], schizophrenia, as well as schizophrenia-related symptoms of insomnia [115].

 Other polymorphisms that may be related to melatonin concern the nuclear receptor genes, ROR*α* (= RORA) and ROR*β* (= RORB = RZR*β*). On the one hand, these receptors are considered as melatonin binding sites [3, 31, 38], but they also represent factors that interact with the circadian core oscillators, sometimes in a tissue-specific manner, and potentially also in a melatonin-independent way [32]. Thus, melatonin-independent actions of ROR*α* or ROR*β* that involve central and peripheral circadian oscillators cannot be distinguished from effects of melatonin solely on the basis of polymorphisms. Because of the latter possibility, and also with regard to effects of melatonin on central and peripheral oscillators [32], pertinent findings shall, at least, be mentioned. ROR*α* polymorphisms were reported to be associated with various subforms of depression [116, 117], though with some methodological limitations (cf. [32]), and macular degeneration [118, 119]. An ROR*β* polymorphism indicated a relationship to bipolar disorder [120]. It will be of future importance to clarify the involvement of altered melatonin signaling in ROR*α* and ROR*β* variants.

## 5. Consequences of Melatonin Deficiency

With regard to melatonin's orchestrating role [1–3, 32], a plethora of effects can be expected to result from its deficiency. The consequences are not only evident in the CNS but extend to numerous other organs. In part, they are related to disturbances of the circadian oscillator system, but additional defects of different nature may also arise.

In the CNS, membrane and nuclear receptors as well as other, poorly investigated putative melatonin binding sites are widely distributed. However, the functional significance is only clear in a few aspects. The most frequently studied role of melatonin concerns the SCN. In mammals including the human, melatonin released from the pineal gland is notably both an output factor steered by the SCN, via a known neuronal pathway and an input factor feeding back to the SCN. These aspects, and especially the roles of melatonin receptors in this hypothalamic structure, have been frequently reviewed, but mostly in relation to the control of the circadian pacemaker [105, 121–124]. A specific effect of melatonin at the SCN is related to sleep. Of course, this action is intertwined with the phase control of the master clock but can be discussed separately, in particular, with regard to sleep initiation. The onset of sleep is favored by MT_1_-dependent actions at the SCN that are further mediated to the hypothalamic sleep switch, a structure that responds in an on-off mode. On the basis of mutual inhibition, it alternately activates either wake-related neuronal downstream pathways that involve locus coeruleus, dorsal raphe nucleus, and tuberomammillary nucleus or, under the influence of melatonin, sleep-related pathways via the ventrolateral preoptic nucleus [125, 126]. However, other brain structures, in which melatonergic receptors are also expressed, are additionally involved. For instance, the thalamus contributes to the soporific effects of melatonin by promoting spindle formation, which is characteristic for the transition from stage 2 sleep to deeper sleep stages and requires a thalamocortical interplay [33, 34, 127]. Although sleep temporally coincides in humans with high nocturnal melatonin levels, persistent effects of melatonin on sleep maintenance are less evident. Nevertheless, low nocturnal melatonin is, independently of its specific causes, generally associated with sleep difficulties [65, 128–131].

Elderly insomniacs exhibit strongly decreased levels and rhythm amplitudes of the excretion product, 6-sulfatoxymelatonin, compared to individuals of same age without sleeping difficulties [128], but this phenomenon is not restricted to individuals of advanced age [3]. In cases of pediatric survivors of craniopharyngioma surgery, the resulting lack of melatonin secretion was associated with inappropriate daytime sleep and nocturnal awakenings [54–56]. A perturbation of the circadian system may contribute to these symptoms but does not fully explain them, since the rhythm of sleep/wakefulness was evident in the actograms [56]. Nocturnal sleep deficits are compensated by the homeostatic drive to sleep and can lead to daytime somnolence as well. Similar changes have been observed after pinealectomy [131], although exceptionally a lengthening of nighttime total sleep duration has also been observed, which was, however, mainly caused by an increased REM sleep duration [132]. Other cases, in which the circadian system was predominantly affected, will be discussed in the next section.

Sleep difficulties are often accompanying symptoms of depressive disorders [133–135]. Chronic insomnia has even been considered as a predictor and, possibly, a triggering factor for this group of diseases [133, 134]. In fact, sleep disturbances have been reported as a prodromal symptom several weeks prior to the recurrence of a depressive episode [133]. However, the etiologic heterogeneity of depressive disorders does not allow to conclude on a general relationship between melatonin deficiency, resulting insomnia, and depression. Nevertheless, this connection may exist in some subforms. Moreover, decreased melatonin levels can be a reason for inefficient entrainment and, thus, inappropriate circadian timing, either with regard to the coupling to external time cues or to internal phase relationships within the multioscillator system [32]. Some types of depressive disorders that are associated with or, possibly, caused by circadian dysfunction will be discussed below.

In addition to its sleep promoting properties, melatonin exhibits various other sedating, antiexcitatory, and anticonvulsant effects, which comprise different actions, such as facilitation of GABAergic transmission, modulation of glutamate receptors, secondary effects by decreases of cytosolic Ca^2+^ via GABA_c_ or metabotropic mGlu_3_ receptors, interference with neuronal NO synthase, changes in K^+^ currents, and potentiation of strychnine-sensitive glycine-induced currents (summarized in [6]). To what extent these functions are impaired under conditions of melatonin deficiency remains to be studied. This may not be obvious, as long as individuals are not challenged by diseases, but can become relevant if excitotoxic and brain inflammatory processes take place, for example, in neurodegenerative disorders or brain infection. Similar considerations may be justified for antihyperalgesic, antinociceptive and anxiolytic effects, which seem to be functionally related to antiexcitatory actions [3, 6, 136].

Numerous other consequences of melatonin deficiency may be deduced from preclinical studies, mainly conducted in nocturnally active rodents. Differences between diurnal and nocturnal species have to be considered especially in all areas concerning neuronal activities, the cardiovascular system, and physical exercise. In these cases, the applicability to humans remains to be demonstrated, especially in the following examples. MT_2_ knockout mice were reported to be impaired in hippocampal long-term potentiation [137], a finding of interest in terms of neuronal plasticity and learning. MT_1_ knockout mice exhibited gradual sensorimotor deficits and increased times of immobility in forced swim tests, which is usually interpreted as an indication of depressed-like behavior [138].

Countless publications have dealt with experimental melatonin deficiency by pinealectomy in animals. Only some studies will be considered here, which are potentially relevant to clinical medicine. In senescent rats, pinealectomy caused enhanced oxidative damage to membrane lipids, protein, and DNA in various organs, compared to controls of the same age [139]. These data are in good agreement with the amply documented antioxidant properties of melatonin, which have been demonstrated in numerous organisms and experimental models [1, 6, 12, 14–17, 19, 25, 140, 141]. Pinealectomy was also reported to increase homocysteine levels, which might indicate a higher risk of cardiovascular disease, results that were in line with the homocysteine-reducing action of melatonin [142]. In models of neurodegeneration, based on focal brain ischemia or glutamate toxicity, the damaged areas were larger in pinealectomized rats than in control animals [143].

The loss of melatonin as a component of the antioxidative protection system may be also relevant during light-induced melatonin suppression in nocturnal shift work [144, 145], in addition to the perturbation of circadian oscillators. Similar assumptions have been made with regard to aging. However, aging is a complex phenomenon during which primary, lingering processes that include increased free-radical formation because of progressive mitochondrial impairments are superimposed by events of deterioration due to diseases, which lead to secondary impairments [19]. Individual catastrophes such as infarction, stroke, renal failure, or cancer impair the function of organs and cells and, thereby, contribute to the acceleration of the more continual mechanisms of aging, even if treatment is successful. Less severe diseases, such as subclinical chronic inflammation, may also affect the health state and lead to a more rapidly progressing senescence. Aging itself and many age-related diseases are associated with increases in free-radical formation, along with a higher vulnerability to oxidative damage and less efficient repair mechanisms [19, 146–152]. On this background, it seems attractive to assume that melatonin, which acts as a direct and indirect antioxidant, improves mitochondrial function, and has some additional cell-protective and antiiflammatory properties, may antagonize senescence. Correspondingly, melatonin deficiency might cause an acceleration of aging and increase the likelihood of developing age-related diseases. However, the direct evidence for this relationship is still insufficient. On the one hand, the health state of experimental animals treated during aging with melatonin is usually better, as referred to as the “Methuselah syndrome” [153]. Old melatonin-treated rodents display a higher mobility, a glossy fur, absence of skin inflammations, and low osteoporosis, compared to age-matched controls [6]. These findings indicate that melatonin deficiency may promote age-related diseases.

On the other hand, life extension by melatonin is not that much apparent in those rodent strains which do not die from cancer [6, 153]. Thus, a longer lifespan observed in mice strains that predominantly die from cancer reflects chemopreventive actions of melatonin rather than a true antiaging effect [6]. However, a prolongation of life by melatonin was demonstrated in the senescence-accelerated SAMP8 mouse strain [154]. These mice are more vulnerable to oxidative stress, but a major question is that of whether the processes responsible for the more rapid aging are identical with the normal causes of aging. As argued elsewhere [19], other progerias, for example, of a laminopathic type, do not reflect normal aging.

Since melatonin is multiply involved in immunodulation [1–3, 6, 9, 155, 156], and since the immune system is deteriorating by age [157–159], melatonin deficiency may also be assumed to contribute to immunological aging. However, the role of melatonin is highly complex in this regard. First, the methoxyindole exerts both immune stimulatory and antiinflammatory properties and can act in a pro- or antioxidant fashion, depending on leukocytes affected and conditions of infection or inflammation. Moreover, melatonin is synthesized by several types of leukocytes [1, 3, 8, 9, 155]. Although many immune cells express melatonin receptors [3, 6, 9, 155] and can, therefore, respond to the circulating hormone, a decrease in melatonin secretion by the pineal gland does not necessarily imply losses of immune functions, as far as they are based on paracrine and autocrine actions of melatonin produced by leukocytes. Nevertheless, the relationship between melatonin and the immune system during aging remains to be an issue worth of future efforts.

 Immunological aspects of melatonin extend to oncological questions. Melatonin deficiency has, in fact, been discussed in terms of the risk of developing certain types of cancer. Oncostatic actions of melatonin have been observed in various experimental models and include growth and apoptosis of malignant cells [160–165]. These findings clearly exceed the immunological role and primarily concern signaling mechanisms in the respective cancer cells. Nonetheless, the development of tumors from transformed cells may be favored under conditions of melatonin deficiency, when immunomodulation by the pineal hormone has declined. In humans, melatonin deficiency has been attributed to a higher incidence of prostate [65], endometrial [75, 166], and breast [65, 167] cancers. It has remained unclear whether the decreases in melatonin have occurred prior to the disease and are contributing factors to tumor development or represent secondary changes induced by the tumor. Moreover, it remains to be clarified whether, or to which extent, the insufficient melatonin levels and the perturbations of the circadian system are decisive.

 An emerging field of melatonin research concerns metabolic disorders, obesity, prediabetic states, diabetes type 2, and general insulin resistance [3, 32, 80, 168]. Numerous data from preclinical studies have shown a regulation of insulin and glucagon release by melatonin. Direct evidence for an involvement of the methoxyindole in diabetes has been obtained in mice, in which insulin resistance is induced by knockout of the MT_1_ receptor gene [169]. Corresponding data were reported for obesity and prediabetic states. Aging rodents showed increases in visceral adipose tissue that were correlated to a decline of melatonin [1]. Weight gain induced in old- or middle-aged rats by high-fat diet, by ovariectomy, or, notably, by pinealectomy was reversed by melatonin, with concomitant normalizations of insulin and leptin levels [170–175]. Similar activities were reported for the synthetic melatonergic agonists NEU-P11 [174] and ramelteon [176]. In humans, the main evidence was obtained in several studies which demonstrated an enhanced risk for diabetes type 2 in variants of the MT_2_ receptor gene (summarized in [32]). Collectively, all pertinent findings indicate that high nocturnal melatonin and intact melatonin signaling are in favor of avoiding diabetes type 2, despite the chronobiological differences between nocturnally active rodents and humans.

 With regard to the numerous sites of melatonin receptor expression within the body, many additional consequences may arise from melatonin deficiency and be clinically relevant. A high number of data, which would by far exceed the scope of this review, exist for effects of melatonin in other tissues, including the cardiovascular system, bones, other endocrine glands, and visceral organs. However, direct evidence, on a clinical basis, for a causative role of melatonin deficiency in their respective diseases would be required for definite judgments.

## 6. Melatonin and Circadian Dysfunction

Circadian dysfunction can result from different causes, such as (i) mutations/variants of circadian core oscillator genes, (ii) impaired light input pathways because of blindness or age-related reduction of photoreception, (iii) inadequate artificial lighting, as far as it is too dim and poor in short wavelengths, or (iv) degeneration of the SCN or the neuronal connections from the retina to the SCN. With regard to peripheral oscillators, epigenetic changes in the promoters of core oscillator genes, as frequently observed in cancer, can likewise lead to local circadian dysfunction [32].

 The role of photoreception requires some distinctions. Visual blindness does not necessarily cause circadian malfunction, especially not if intact melanopsin-containing retinal ganglion cells and the connection to the SCN are retained. On the other hand, circadian photoreception may significantly decrease in the aging eyes of sighted persons because of pupillary miosis and reduced crystalline lens transmission in the short wavelength range. This can already lead to circadian disruption and increase the incidence of sleep problems, affective disorders, metabolic syndrome, and other systemic diseases [177].

 Physiologically unfavorable deviations of rhythmicity can be identified at the level of the circadian oscillators, their phasing by external time cues, and their internal coordination within the multioscillator system. These alterations occur under the following conditions [32, 178, 179]. (i) Reduction in the amplitude of a single oscillator, for example, during aging and under weak zeitgeber strength, especially in persons with visual impairments, night-eating syndrome, or insulin resistance. A flattened oscillation has a reduced capability of interacting with other oscillators, which results in the loss of stable internal phase relationships. Moreover, the coupling to external time cues may be impaired. (ii) Phase shifts that lead to internal circadian disruption if the synchronizing signal affects the various oscillators differently. If a light-sensitive oscillator is reset under conditions of rotating shift work or light at night because of other reasons, other oscillators that are synchronized by the onset of darkness or by nonphotic time cues may not follow and become uncoupled. (iii) The central pacemaker is poorly synchronized with the external cycle, as known under the term “free-running disorder” (FRD). Deviations in the spontaneous period of the central pacemaker can either lead to a nonsynchronized free-running oscillation or to a poorly coupled rhythm that shows relative coordination with the environmental cycle. Additionally, the internal coordination within the multioscillator system may be disturbed. FRD may be either caused by impaired photoreception, as observed in some blind individuals [180–185], or by genetic predispositions. Despite the existence of data from animals related to mutations in clock genes and other factors affecting oscillators, the knowledge on respective human polymorphisms is limited. However, genetic causes may be deduced from the existence of FRD in sighted individuals with a disease onset during youth [186–188]. In humans, more is known about the genetic basis of extreme morningness or eveningness, symptoms that are associated with pronounced deviations of the spontaneous, free-running period observed in the absence of external zeitgebers. In these cases, synchronization with external time cues is still possible, but the phasing of rhythms deviates from normal and results in circadian rhythm sleep disorders (CRSDs). Polymorphisms in the core oscillator genes *Per2* and *Per3* (*period 2* and *3*) have been identified as being causative of some CRSDs [187, 189], but mutations in other clock genes may lead to similar changes as well.

 The coupling strength of oscillators within the organism differs between individuals. This is already evident in the classic cases of humans investigated in isolation. A certain number of subjects deprived from external time cues shows the phenomenon of internal desynchronization, as first described by Aschoff and Wever [190], whereas others do not. The desynchronization is reflected by strongly deviating spontaneous periods of the sleep/wakefulness and body temperature rhythms. However, the coupling between the two oscillators is not entirely abolished, since they show relative coordination, that is, they partially attract each other for a few cycles but, thereafter, dissociate again. In these individuals, the internal coupling force is too weak to maintain robust coordination under free-running conditions. Deviations in coupling of free-running oscillators are even normally observed in individuals without internal desynchronization. In these cases, the phase difference between sleep/wakefulness and temperature rhythms is altered, although both clocks oscillate with the same period.

 The interindividual variations indicate that internal uncoupling may more easily occur in some subjects than in others, when rhythms are either perturbed or weakened. Malcoordination of oscillators within the organism's complex clockwork seems to be of pathological relevance, when this occurs under conditions of normal life. This may play a role in some disorders, in which infradian [191] cycles, that is, rhythms with periods of several or many days, become apparent in addition to other, physiologically normal infradian rhythms (such as circaseptan or circatrigintan cycles [191]). The atypical long cycles, which result from relative coordination of weakly interacting oscillators, are particularly observed in mood and performance. In practice, however, these long cycles are difficult to distinguish from poor synchronization of a single oscillator to an external time cue, which would reflect relative coordination between endogenous and zeitgeber cycles. In any case, physiologically atypical infradian rhythms are indicative of disturbances in the circadian oscillator system. Moreover, a weak coupling of an oscillator to the external cycle may already suffice for the development of symptoms if the rhythm remains synchronized but is strongly dysphased. Alterations of that type are likely present in bipolar and seasonal affective disorders [192–196], assumptions that have gained support by epidemiological studies on core oscillator gene polymorphisms (summarized in [32]). Changes of this kind can be expected to result in sleep difficulties. However, the reverse conclusion that sleep disturbances are indicative of a malfunctioning circadian system is not generally possible [197]. In major depressive disorder, which is typically associated with sleep problems, no convincing evidence exists for the involvement of the circadian system [198], although this may not be entirely excluded in some subtypes.

 Changes in the circadian multioscillator system may be sometimes rather cryptic but become evident by molecular analyses of the rhythmic expression of core oscillator genes. It is important to be aware that multiple oscillators do not only exist in different organs but also in the same organ, in which parallel oscillations are based on the alternate expression of orthologs or paralogs of the clock genes [32]. Again, the coupling between these parallel oscillators can be affected by pathophysiological or, in animal models, experimental disturbances. These approaches provide valuable evidence for the importance of melatonin in the internal coordination of rhythms. In the rat SCN, pinealectomy leads to an abnormally large phase difference between the parallel oscillations in the expression of the alternate core oscillator genes *Per1* and *Per2*. This change is reversed by melatonin administration [199]. The relevance of melatonin has also been demonstrated in another autonomous circadian oscillator of the CNS, the murine retina. In the melatonin-proficient mouse strain C3H, prominent oscillations were observed in the core oscillator proteins PER1 and CRY2 (cryptochrome 2) as well as in CREB phosphorylation, whereas a significant rhythmicity of these factors was absent in the melatonin-deficient mouse strain C57BL [200]. Moreover, knockouts of either MT_1_ or MT_1_ and MT_2_ produced considerable phase shifts in the circadian rhythm of PER1, without abolition of the rhythm itself [201].

In the autonomous clock of the adrenal cortex, PER1, CRY2, and BMAL1 (brain and muscle ARNT-like 1; alias ARNTL) protein levels oscillated with substantial amplitudes in the melatonin-proficient mice, whereas the deficient animals did not exhibit robust rhythms in these core oscillator proteins [202].

In cultures of murine striatal neurons, the knockout of the melatonin receptor MT_1_ abolished melatonin-induced changes in the expression of various core oscillator genes [203]. In cultured mouse cerebellar granule cells treated with nanomolar concentrations of melatonin, the deletions of either MT_1_ or MT_2_ caused losses in the inhibition of forskolin-stimulated cAMP synthesis and cFos expression, as well as in the inhibition of Akt (= PKB) activation. These knockouts were also reported to turn the normally observed suppression of the MAP kinase ERK into an upregulation [204].

In humans, the chronobiological role of the pineal hormone became also evident in several studies. A child with congenital melatonin deficiency exhibited a non-24-hour sleep-wake rhythm, which was corrected by melatonin treatment [205]. An entirely different case of melatonin deficiency was caused by a defect mutation in the sepiapterin-reductase gene [206]. In this patient, serotonin synthesis was impaired and the poor precursor availability prevented the formation of substantial amounts of melatonin. Although serotonin has its own role in sleep regulation, the symptoms of hypersomnia, a circa 12-hour sleep-wake rhythm, and hyperphagia, were normalized by melatonin treatment and were, thus, attributed to the deficiency of the pineal hormone [206].

Another congenital disorder, the Smith-Magenis syndrome (SMS), is not only associated with developmental and neurobehavioral abnormalities but also causes an almost inverted melatonin rhythm and sleep difficulties [207, 208]. SMS is caused by haploinsufficiency of the transcription factor RAI1 (retinoid acid induced 1) [209, 210], whose relationship to melatonin and circadian rhythmicity is unclear. At least, the sleep-related symptoms have been alleviated by suppressing melatonin synthesis during the day by a *β*_1_-adrenergic blocker and melatonin administration in the evening [211, 212].

Collectively, all these findings indicate that melatonin is required in multiple ways in adjusting circadian rhythms, by providing a feedback to the SCN and also by safeguarding phase relationships between parallel oscillators in the SCN and between various autonomous clocks of the multioscillator system. This conclusion is further supported by a number of reports on effects of melatonin on the expression of core oscillator genes and on resetting of rhythms in various peripheral tissues, as recently summarized [32].

## 7. Options for Treatment

With regard to treatment, it is important to distinguish between disorders in which only short actions of melatonin are required and those in which a substitution therapy aims to replace insufficient nocturnal levels of the hormone over an extended period throughout the night. The necessity of this distinction results from the short half-life of melatonin in the circulation, which is, depending on time of administration and other factors, in the range of 20–45 min [35, 213]. Extended melatonergic actions are, therefore, only possible with prolonged release formulations of the natural hormone or with synthetic, longer-acting agonists.

 Only a short action of melatonin is required in the case of sleep onset difficulties. Melatonin is known to reduce sleep onset latency, frequently determined as LNA (latency to nonawake) or, by polysomnography, as LPS (latency to persistent sleep). Both melatonin and all efficacious melatonergic agonists exhibit this property [35, 197, 214]. In contrast to recommended doses of synthetic agonists, such as 4 or 8 mg/d (ramelteon) and 25 mg (agomelatine), melatonin promotes sleep onset already at doses of 0.1–0.3 mg/d of an immediate release formulation [215]. Consequently, melatonin should be preferred as long as no additional effects are intended, such as support of sleep quality or maintenance. The natural hormone has without any doubt, advantages in terms of tolerability, physiological metabolism and, perhaps, MT_1_/MT_2_-independent effects that should be absent in the synthetic drugs [7, 197].

 Another area in which short actions are sufficient concerns the chronobiotic, that is, phase-shifting properties of melatonin. Resetting of the circadian oscillators is recommended or even a requirement in the case of rhythm perturbations. These may have been induced either (i) externally by light at night or transmeridian flights or (ii) by clocks poorly coupled to the environmental cycle or internally to other clocks, in cases of dysphasing or desynchronization within the multioscillator system. Insufficient coupling may result from flattened oscillations, especially under conditions of reduced melatonin secretion due to age or disease. Phenomena such as relative coordination, internal desynchronization, and abnormal phase relationships between parallel oscillators have been poorly investigated on a systematic basis, but they seem to be involved in impairments of physical and mental fitness as well as bipolar and seasonal affective disorders [192–196]. As far as circadian malfunctioning is implicated in these mood disorders, melatonin can be effective in readjusting rhythms and, thereby, improving symptoms. Similar treatments with synthetic melatonergic drugs can be expected to be likewise beneficial, but neither a higher receptor affinity nor a longer half-life is a reason for assuming a superior efficacy compared to melatonin.

Circadian oscillators are largely sensitive to the so-called nonparametric resetting stimuli [216], that is, changes of sufficient extent rather than absolute levels of the synchronizer. Therefore, a short-acting drug can meet the requirements for phase adjustments. Immediate-release formulations of melatonin should be suitable, and a need for using synthetic agonists instead is not apparent. However, such a treatment has to consider some fundamental chronobiological rules. Resetting signals are acting according to a phase response curve (PRC). PRCs describe the dependence of a phase shift on the circadian phase of stimulus administration. Typically a PRC contains an advance part, a delay part, and a silent zone, in which the same signal, which otherwise causes advances or delays of the rhythm, has no or only minor effects. In humans, the PRC for melatonin is known [36, 37]. The time of melatonin administration according to the PRC is of utmost importance. Readjustment of rhythms by melatonin will only be achieved if it is given in an appropriate, sufficiently sensitive phase within the circadian cycle. If the rhythm is dysphased because of poor coupling to synchronizers, it may take several days more until the oscillation has attained the desired phase. Disregard of these chronobiological fundaments may lead to false conclusions on inefficacy.

 It should also be noted that the circadian rhythms can be efficiently reset by light, as long as light perception in the blue range and neuronal connections to the SCN are not impaired. Light therapy is, thus, an option in these cases, whereas, under conditions of poor accessibility of the SCN to light signals, melatonin may be preferred. In some individuals, a combination of light and melatonin in the different, respective phases may be suitable as well. Apart from jet lag, attempts of resetting the circadian oscillators by melatonin and/or light may be especially successful in cases of bipolar and seasonal affective disorders, as far as they can be attributed to rhythm perturbations [32, 192–196]. Whether or not melatonin has an additional, MT_1_/MT_2_-independent effect not exerted by synthetic agonists remains to be clarified. A polymorphism of the ROR*β* (= RORB = RZRB) gene has been attributed to a risk of bipolar disorder [120]. Melatonin is believed to act as an ROR*β* ligand [38], and ROR*β* knockout mice showed atypical PRCs and a lengthened circadian period [217, 218], but the possibility exists that ROR*β* acts on the circadian core oscillator in a melatonin-independent way [32]. Since melatonin phase-shifted circadian rhythms in the ROR*β* knockouts, though in an atypical manner, Masana et al. [218] had concluded that melatonin is not involved in the deviations because of ROR*β* deficiency.

 The situation is entirely different if a replacement therapy is desired in aged individuals or patients suffering from diseases that are associated with decreases in melatonin levels. Because of the short half-life, immediate-release formulations of melatonin cannot afford a satisfactory substitution. With regard to melatonin's superior tolerability, a prolonged-release tablet of the pineal hormone may be tested first [35], such as Circadin, which is approved in Europe by EMEA for the treatment of insomnia in patients aged 55 years and over. If the improvements are too poor, longer-acting synthetic MT_1_/MT_2_ agonists may be tested. To date, two drugs of this type have been licensed, ramelteon (Rozerem = TAK-375) for treatment of insomnia in the USA, and agomelatine (Valdoxan) for treatment of major depressive episodes (MDE) in adults in Europe. Ramelteon has a higher affinity than melatonin to MT_1_ and MT_2_ receptors and is devoid of relevant affinities to other receptors, whereas agomelatine exhibits similar affinities to MT_1_ and MT_2_ as melatonin but acts additionally as a 5-HT_2C_ antagonist, a property attributed to its antidepressive actions. Both drugs are more slowly eliminated from the circulation than melatonin and are, thus, longer acting. For comparison of affinities, pharmacokinetics, metabolism, and efficacy of melatonin, ramelteon, and agomelatine, see [35].

 A few remarks seem to be necessary concerning the use of the synthetic melatonergic agonists. First, the therapeutic limits of the respective licenses should be considered, which excludes some uncontrolled treatments. Moreover, the deviating characteristics of the synthetic compounds are of importance, especially with regard to metabolism and drug interactions. The metabolism of ramelteon leads to several compounds, one of which has unusual properties. This metabolite, referred to in literature as M-II, has affinities to MT_1_ and MT_2_ of about 10% of ramelteon, but a 2–5 h longer half-life. It can attain concentrations by 20- to 100-fold higher than the parent compound [35, 215, 219, 220] and, thus, contributes substantially to the overall effect of the drug. Ramelteon is mainly metabolized by CYP1A2, CYP2C9, and CYP3A4, and, consequently, other drugs that inhibit one or several of these isoenzymes, such as fluvoxamine, ciprofloxacin, mexiletine, norfloxacin, azileuton, fluconazole, or ketoconazole, have to be avoided. For other conditions of concern, see [215]. Although ramelteon has a higher affinity to the melatonin membrane receptors and although it acts longer than melatonin, the recommended doses of 4 or 8 mg/day are higher than those of melatonin prolonged-release (2 mg/d). Like melatonin and other melatonergic drugs, it reduces sleep latency. With regard to sleep maintenance, as tested in elderly patients with primary chronic insomnia, the improvements by ramelteon were found in a recent review to be highly variable [221]. Despite some statistically demonstrable increases in sleep duration or sleep efficiency, this does not yet imply complete restoration of persistent sleep throughout the night [221–223]. Prolonged-release melatonin was rather poorly effective on sleep duration in this type of patients [35], although sleep quality, next-day alertness, and measures of quality of life were moderately improved. In view of these results, one might conclude that both ramelteon and prolonged-release melatonin are of some practical value, but that a sufficient replacement therapy has not yet been achieved.

During the last years, the necessity of long-term studies for ramelteon has been expressed, especially with regard to hepatotoxicity, micronuclei formation, and mutagenicity. This should include the metabolite M-II, which attains concentrations in the range of one third of the no-effect level for tumor induction of the parent compound [215, 220]. Several recent papers indicate long-term safety of ramelteon in their titles [224–226], but they mainly address adverse events such as nausea and headache, some hepatological parameters, lack of residual effects, of rebound insomnia, of withdrawal symptoms, and dependence for periods of six or twelve months, but not the absence of mutagenic/carcinogenic actions over extended treatment.

 Agomelatine, which has been licensed only for treatment of major depressive episodes, displays all the sleep-inducing and chronobiotic effects known from melatonin [61, 134, 135, 227]. With regard to these properties, it seems similarly suitable as ramelteon or melatonin. However, the focus on depressive symptoms leads to problems in justifying treatment and also to some misunderstandings. Again, a distinction between types of depression based on circadian dysfunction or on other reasons is important. In the first case, the efficacy of agomelatine cannot be distinguished from those of other melatonergic agonists of similar receptor affinity. However, in major depressive disorders, the improvements of symptoms have been attributed to the inhibition of 5-HT_2C_ receptors [134, 135, 220, 227–230]. However, this property is not comparable to effects by classic antidepressants. For this reason, some authors have considered the efficacy of agomelatine insufficient in major depressive disorder [231–233]. Moreover, biased publication on the efficacy of this drug has been criticized [234]. However, it should be noted that the advantages of agomelatine do not consist in a superior antidepressive effect, but rather in the combination of antidepressive benefits with sleep improvements, which is important because sleep disturbances are often induced by classic antidepressants [134, 135, 220, 230]. Agomelatine, being a naphthalene derivative, may also become a matter of concern under aspects of toxicity. Although it is well tolerated according to adverse events during short treatment, absence of rebound insomnia, withdrawal symptoms, and dependence, we had previously emphasized that a naphthalenic compound requires thorough studies on long-term toxicity, including CYP-related hepatic effects and, eventually, carcinogenicity [35, 197, 220]. This is not so much a question of drug interactions with CYP1A2 inhibitors, which have to be avoided in any case, but rather of the formation of toxic naphthalenic metabolites. Under this aspect, the high recommended dose of 25 mg/d may turn out to be a disadvantage of agomelatine. This issue has been recently readdressed. Moderate increases in liver enzymes are rather common, but serious hepatotoxicity is rare during treatment of moderate length [231, 233]. It was also criticized that the risk of hepatotoxicity had not been prominently documented in the published studies [232, 234], and oncogenicity of very high doses in animals experiments [232] should be taken as a caveat. It should also be noted that agomelatine, being a melatonergic agonist, can only be administered, for chronobiological reasons, in the evening. Conclusions on antidepressive effects cannot be convincingly drawn from studies conducted in nocturnally active rodents, in which melatonin is associated with the dark phase and activity. This reservation is valid for all melatonergic drugs but has been disregarded by some investigators in models of depression and anxiety, and even in studies on sleep (for criticism, see [197]).

 In addition to the licensed products, numerous investigational drugs have been developed, some of which may have advantages and may be approved in the future [197]. Among these, *β*-methyl-6-chloromelatonin (= TIK-301 = LY 156735), tasimelteon, GR 196429, the indane compound GG-012, several substituted 8-methoxytetralins, double ring compounds like UCM765 and UCM924, in which halogen atoms are positioned to avoid CYP-dependent hydroxylations, and NEU-P11 may be mentioned (for comparisons, see [197]). The suitability of these compounds will depend on nontoxicity and superior efficacy compared to other drugs. Receptor affinities are sometimes in the range of melatonin, but not rarely considerably lower, which would require very high doses. Advantages are mostly observed in a half-life much longer than that of melatonin.

 One of these newly-developed drugs, NEU-P11, has been preclinically studied in another area, which may gain importance in the future, namely, metabolic disorders including obesity, insulin resistance, and diabetes type 2 [174]. The significance of melatonin in this complex of disorders and diseases has become particularly evident during recent years [3, 32, 80, 168]. A disadvantage of NEU-P11 may be seen in its relatively low receptor affinity, whereas its half-life is favorable. In this area, future comparisons with other melatonergic drugs will be of interest.

## 8. Conclusion

Melatonin deficiency is common in aging and also associated with various diseases of different etiology. Moreover, melatonin secretion is suppressed by nocturnal light, which can be a matter of concern in shift work. With regard to the multiplicity of melatonin's actions, the consequences of melatonin deficiency extend to numerous physiological functions. If subnormal melatonin levels are caused by neurodegeneration in the SCN, which controls the mammalian pineal gland and to which this hormone is feeding back, administration of the methoxyindole cannot readjust the circadian oscillator system in full. Effects on some peripheral oscillators are not excluded, but this has not yet been demonstrated under such conditions. Effects on other physiological functions that are accessible independently of the circadian oscillator system, but not necessarily devoid of normally occurring circadian oscillations, may still be positively affected by melatonin. Examples may be found in the areas of immune modulation, antioxidative protection, correction of metabolic syndrome, or gastrointestinal functions.

 Melatonin dysfunction can result from different causes. Melatonin receptor variants, which have been found to be associated with risks for various diseases [3, 32], presumably lead to impaired or incomplete signal transduction. However, the cell biological details of these changes remain to be elucidated. Dysfunctions can also arise from abnormalities of the circadian oscillator system, because of either polymorphisms of core oscillator genes that lead to deviatant period lengths of the rhythms or impaired light input pathways towards the SCN. Again, gene variants causing exceptionally long or short periods are associated with risks for various diseases [32].

 Successful treatment of melatonin-related disorders largely depends on the following criteria. (i) If only short actions of melatonin are required, improvements are likely. This is the case in sleep initiation and in disorders with an etiology of poor synchronization by external time cues and, perhaps, those characterized by poor internal coupling within the multioscillator system. This phenomenology seems to be of relevance in seasonal effective and bipolar disorders. (ii) Since the SCN is involved in the entrainment by melatonin, the structural and functional integrity of this hypothalamic structure has to be sufficiently preserved. (iii) Melatonin receptors have to be expressed at fairly normal levels and signal transduction should not be impaired because of mutations in the receptor genes.

 All the treatments that only require short actions of melatonin can be successful, under conditions mentioned, by using relatively moderate amounts of immediate-release melatonin. Although synthetic melatonergic agonists are likewise effective in sleep initiation and improvements of circadian rhythm-related disorders, a need for their use or a substantial superiority is not evident. The higher recommended doses of the synthetic drugs and the remarkable tolerability of the natural hormone are clearly in favor of melatonin. Nevertheless, several contraindications or possible reasons of concern should be considered prior to prescription, such as use in children—except of severe or otherwise intractable cases, during pregnancy, in patients with autoimmune diseases, liver dysfunctions, medication with CYP1A2 inhibitors [35], and, unless the issue is definitely settled, Parkinson's disease and irritable bowel syndrome type II, which have both been interpreted as diseases of melatonin overproduction (“melatonin hyperplasia”) [235].

 For treatment of disorders that require extended melatonergic actions, melatonin prolonged release formulations and synthetic longer-acting drugs have been developed. If judged according to criteria of sleep maintenance, some improvements can be achieved especially by synthetic agonists such as ramelteon. Even though these effects are statistically demonstrable in some studies, this is not generally so. More importantly, it has to be stated that the improvements remain rather moderate in their extent, even if they are significant according to formal statistical criteria. The somewhat disappointing conclusion is that a full replacement therapy of melatonin deficiency, which restores high melatonergic activity throughout the night, has not been successful to a desirable degree. Nevertheless, further studies may reveal improvements in other physiological parameters not primarily or directly related to sleep, such as measures of lipid metabolism, mitochondrial function, or sensitivity to insulin. Whether or not synthetic drugs will turn out to be superior to melatonin in these fields remains to be demonstrated.

## Figures and Tables

**Table 1:** Diseases and disorders that cause decreases in human* melatonin secretion independently of apparent tissue destruction in SCN or pineal gland.

Disease/disorder	Comments	References
Schizophrenia	Only in a subpopulation	[57, 58]
Multiple sclerosis with major depression	In major depression alone, mostly phase shifts instead of decreases	[59–62]
Primary obsessive-compulsive disorder		[63]
Menière's disease	Relationship to stress by tinnitus and vertigo?	[64]
Fibromyalgia	Studied in women	[65–67]
	Pain reduced by melatonin	[67–69]
Migraine		[70, 71]
Critical illness		[72–74]
Endometrial cancer		[75]
Nonsmall cell lung cancer	In part caused by pain?	[76]
Acute intermittent porphyria	Further decreased by seizures	[77, 78]
Diabetes type 2		[79, 80]

*For preclinical findings of these and other diseases or respective animal models see [3].
